# Comment on: Is the absence of Right Hepatic Vein opening into Inferior Vena Cava a contraindication for right lobe liver donation in Living Donor Liver Transplantation? Common hepatic venous trunk—A rare hepatic veinanomaly: A case report and review

**DOI:** 10.1016/j.ijscr.2019.11.069

**Published:** 2020-01-10

**Authors:** Sami Akbulut

**Affiliations:** Liver Transplant Institute, Inonu University Faculty of Medicine, 244280, Malatya, Turkey

To the Editor

We read with great interest the recent article “Is the absence of Right Hepatic Vein opening into Inferior Vena Cava a contraindication for right lobe liver donation in Living Donor LiverTransplantation? Common hepatic venous trunk—A rare hepatic vein anomaly: A case report and review” published by Ray et al. [1]. The authors stated that the right hepatic vein did not opened directly to the inferior vena cava, but instead the right hepatic vein first jointed with the middle hepatic vein and left hepatic vein, and then the formed trunk was opened directly to the inferior vena cava. We would like to share our opinion and criticisms about this valuable work as follows.

First, the authors present the presence of inferor right hepatic veins as a vascular anomaly. We strongly disagree with this opinion of the authors. According to my experience from a liver transplant center, more than half of both recipients and donors have one or more inferior right hepatic veins, which is supported by the literature [2,3]. Therefore, it is not a correct approach to consider structures seen at such a high rate as deviations from normal.

Secondly, the authors no provided any information about back-table venous drainage reconstruction model, graft implantation techniques, and whether the recipient experienced any complications related to postoperative venous drainage.

Thirdly, the presence of variations or anomalies in the vascular structures of the liver and their incidental detection during radiological examinations mean nothing in clinical terms. Because these patients are often asymptomatic. In our opinion, the most important issue is how to proceed if vascular anomaly or variation is detected in living liver donor and whether these donor candidates will be refused. The answer to these questions depends on whether the recipient has a chance to find another living liver donor and the transplant center's experience on vascular reconstruction.

When the title of the study is examined, it is seen that the article is prepared as case report and literature review. Moreover, when the text of the article is examined, it is understood that such an anomaly has not been identified in the living liver donor before. However, this is not true. The first case of congenital right hepatic vein absence in the living liver donor was reported by our team [4].

## Declaration of Competing Interest

No conflict of interest about this letter to the editor.

## Sources of funding

No received funding.

## Ethical approval

No required ethical approval.

## Consent

This work was written in the form of ”letters to the editor”. Therefore, no patient was presented.

## Author contribution

This letter was designed and written by Akbulut.

## Registration of research studies

No.

## Guarantor

No.

## Provenance and peer review

Editorially reviewed, not externally peer-reviewed.
